# Supplementary material for: 1,5,6-Trimethoxy-2,7-dihydroxyphenanthrene from Dendrobium officinale Exhibited Antitumor Activities for HeLa Cells
Source: Int J Mol Sci. 2023 Oct 19;24(20):15375. doi: 10.3390/ijms242015375 (PMC10607032; doi:10.3390/ijms242015375)
Supplement: Supplementary file 1 [file ijms-24-15375-s001.zip › ijms-2587315-supplementary.pdf]

Supplementary Material

1,5,6-Trimethoxy-2,7-dihydroxyphenanthrene from *Dendrobium officinale* Exhibited Antitumor Activities for HeLa Cells

Chong Liang [1], Chonglun Zhang [2], Yinlin Zhuo [1], Baocheng Gong [1], Weizhuo Xu [3,*] and Guogang Zhang [1,*]

[1] School of Traditional Chinese Materia Medica, Shenyang Pharmaceutical University, 103 Wenhua Road, Shenhe District, Shenyang 110016, China; liangchong7628@163.com (C.L.); 18750097073@163.com (Y.Z.); gkcmu_1h@163.com (B.G.)
[2] School of Life Sciences and Biopharmaceuticals, Shenyang Pharmaceutical University, 103 Wenhua Road, Shenhe District, Shenyang 110016, China; zhangcl0630@163.com
[3] School of Functional Food and Wine, Shenyang Pharmaceutical University, 103 Wenhua Road, Shenhe District, Shenyang 110016, China
* Correspondence: weizhuo.xu@syphu.edu.cn (W.X.); zggth@163.com (G.Z.); Tel./Fax: +86-024-43520301 (W.X.); +86-024-43520735 (G.Z.)

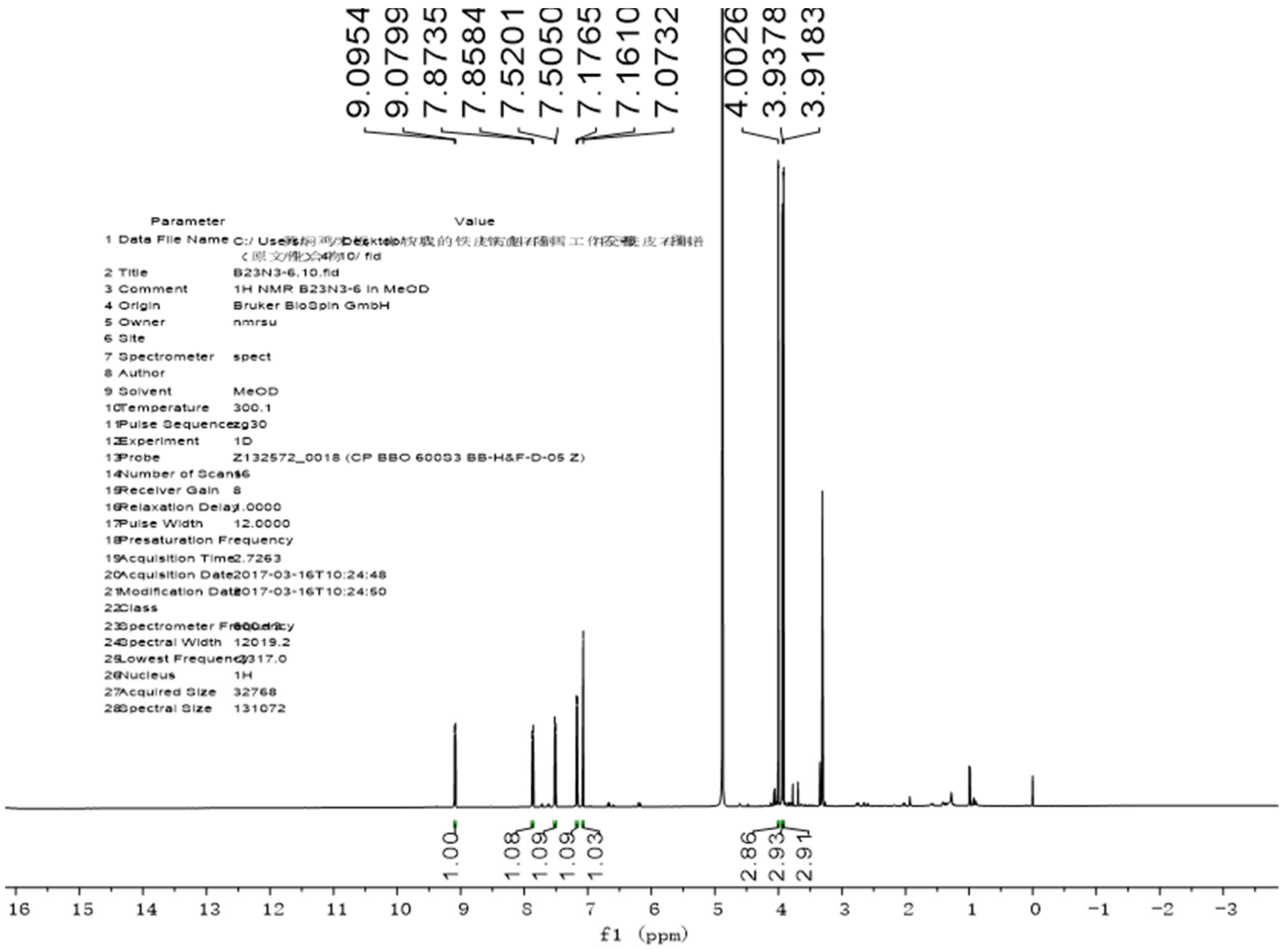

Figure S1. ^{1}H NMR spectrum for compound 5.

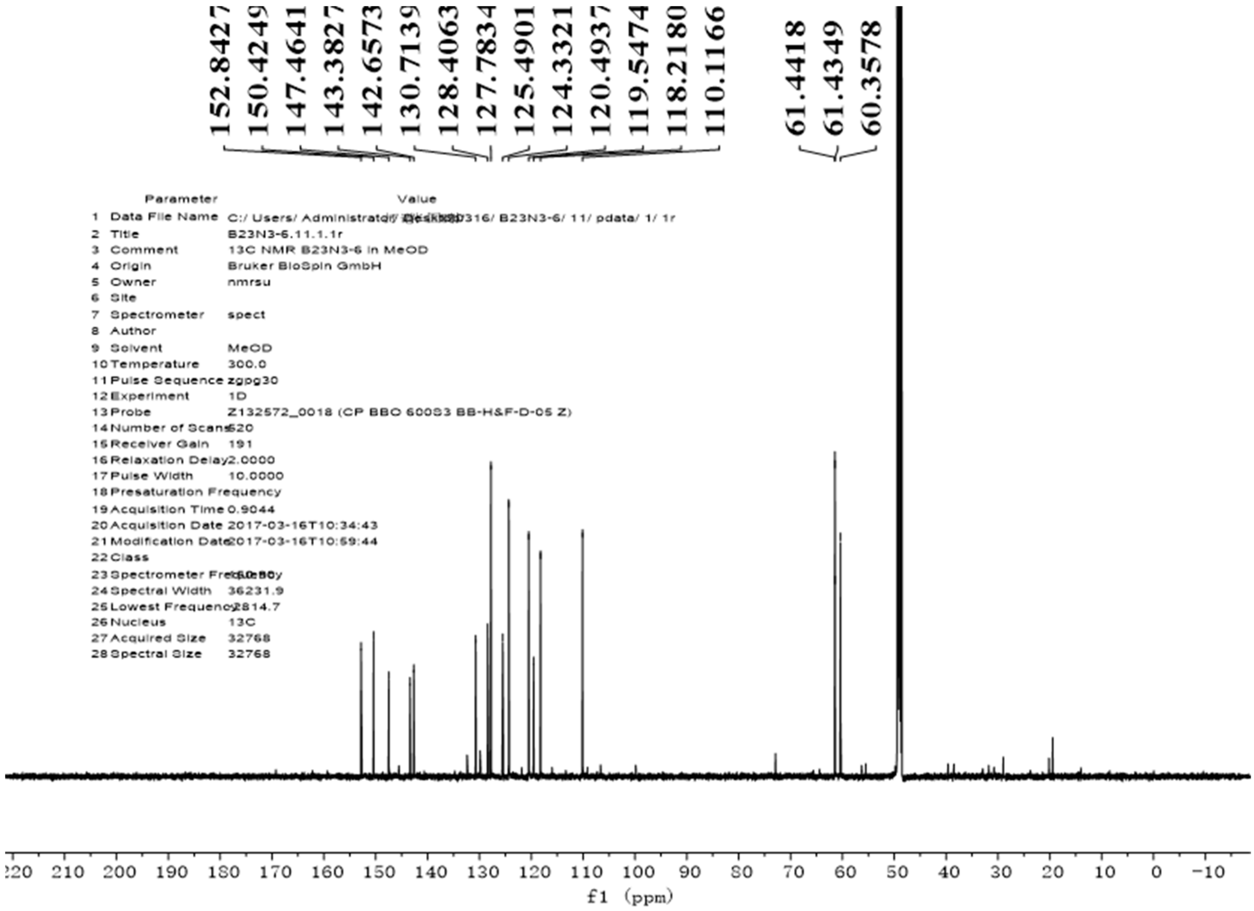

Figure S2. ^{13}C NMR spectrum for compound 5.

	DMSO	3.125μM	6.25μM	12.5μM	25μM	50μM	100μM
the first time	0.577	0.161	0.148	0.114	0.116	0.111	0.126
	0.534	0.171	0.144	0.119	0.115	0.109	0.128
	0.498	0.163	0.131	0.113	0.116	0.109	0.134
the second time	0.543	0.303	0.189	0.152	0.123	0.1	0.105
	0.714	0.352	0.226	0.171	0.129	0.11	0.109
	0.599	0.303	0.213	0.157	0.135	0.097	0.111
the third time	0.647	0.318	0.206	0.161	0.12	0.102	0.115
	0.636	0.308	0.185	0.123	0.114	0.097	0.105
	0.618	0.243	0.147	0.12	0.133	0.099	0.114

Figure S3. OD value of compound 5 at 490nm.

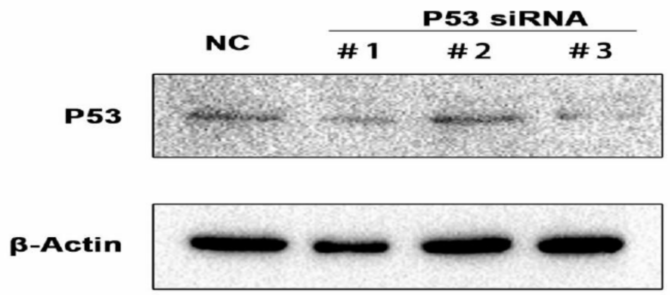

Figure S4. Expression of p53 after siRNA treatment
